# Breakfast Consumption in Spain: Patterns, Nutrient Intake and Quality. Findings from the ANIBES Study, a Study from the International Breakfast Research Initiative

**DOI:** 10.3390/nu10091324

**Published:** 2018-09-18

**Authors:** Emma Ruiz, José Manuel Ávila, Teresa Valero, Paula Rodriguez, Gregorio Varela-Moreiras

**Affiliations:** 1 Spanish Nutrition Foundation (FEN), C/General Álvarez de Castro 20, 1 pta, 28010 Madrid, Spain; eruiz@fen.org.es (E.R.); jmavila@fen.org.es (J.M.Á.); tvalero@fen.org.es (T.V.); prodriguez@fen.org.es (P.R.); 2 Department of Pharmaceutical and Health Sciences, Faculty of Pharmacy, CEU San Pablo University, Urb. Montepríncipe, Crta. Boadilla Km 53, Boadilla del Monte, 28668 Madrid, Spain

**Keywords:** breakfast consumption, dietary quality, ANIBES Study, NRF 9.3, Spanish diet, food groups

## Abstract

This study aimed to investigate energy, nutrient and food group intakes at breakfast in Spain and to examine for the first time, their relationship to the overall Diet Quality (DQ). The data used were from the Spanish ANIBES (anthropometric data, macronutrients and micronutrients intake, practice of physical activity, socioeconomic data and lifestyles in Spain), a cross-sectional study using a nationally representative sample of the Spanish population (9–75 years old). DQ was assessed using the Nutrient Rich Foods Index, adapted to total diets (NRF9.3d). Most (>85%) of the Spanish population were regular breakfast consumers, although one in five adolescents were breakfast skippers. Breakfast provides just 16–19% of the daily intake of energy. Relative to its daily energy contribution, the Spanish breakfast contributed a higher proportion of daily total carbohydrates, added sugars, sodium, thiamin, riboflavin, folates, iron, potassium, magnesium, phosphorus and especially in calcium. By contrast, the breakfast is low in water intake, protein, dietary fibre, total fat, polyunsaturated fatty acids, beta-carotene and vitamins E and D. In children and teenagers, the most commonly consumed breakfast food was chocolate (mainly as chocolate-flavoured milk and powder), followed by bakery and pastry, whole milk and semi-skimmed milk. In the older groups, a bigger variety of foods were reported. Consumers in the highest NRF9.3d tertile for diet quality tended to have a higher intake of positive nutrients at breakfast than other tertiles, most notably among adults.

## 1. Introduction

Spain has undergone dramatic social and socioeconomic changes since the 1960s, including massive rural–urban migration, rapid urbanization processes during the 1980s and the generalized incorporation of females into the active workforce [1]. As a result of these transitions, the Spanish population has partially turned away from its traditional Mediterranean diet, including dietary patterns across the day, which has mainly occurred in the youngest segment of the population. The changes in diet, physical activity and lifestyle seem to have had potentially negative consequences for the both present and future populations. Overweightness and/or obesity affect more than 50% of the adult population and nearly 30% of infants and children [2,3].

There is general agreement that breakfast should play a significant role in helping consumers to attain better dietary and nutritional profiles, as well as healthier lifestyles [4,5,6]. The breakfast most frequently consumed in Spain consists of a dairy product, particularly skimmed or semi-skimmed milk and cereal, especially bread, which may differ from the findings in other European populations [7,8,9]. Dietary recommendations in Spain suggest that a healthy and a nutrient density-adequate breakfast should contribute around 20–25% to the total daily energy intake [7,10,11] and foods should be selected at least from three key food groups, namely: starchy foods (cereals, pasta, bread), fruit and vegetables and milk and dairy products [7,11]; additionally, healthy fats (e.g., virgin olive oil) may be also an adequate complement. Some recent and varied findings in the Spanish population highlight the role of breakfast in public health and the need for its monitoring and promotion. Therefore, skipping breakfast has been associated with increased odds of prevalent non-coronary and generalized atherosclerosis in Spanish adults (40–54 years), independent of the presence of conventional cardiovascular risk factors [12]. In the Spanish adult population (18–64 years), habitual breakfast consumption was negatively associated with abdominal obesity [13]; a higher percentage of people ate more than four meals daily (including breakfast) and there was a higher rate of consumption of cereals and dairy products in the group without central obesity [2]. Interestingly, regular breakfast consumption in adolescents from the HELENA Study (including Spain) was associated with higher blood vitamin D and cobalamin concentrations in males and with higher vitamin D and holo-transcobalamin and lower tHcy (total homocysteine) concentrations in females [14]; moreover, breakfast consumption was associated with high intakes of vitamin D and total folate in both sexes and with high intakes of vitamin B6 and vitamin E in females. It should be also highlighted that an updated report on the breakfast situation in Spain has been published jointly with the first edition of National Breakfast Day, to create a greater awareness among the population, not only about the importance of having breakfast but also on its quality [7]. One of the key conclusions of the latter was the need to have national representatives and updated information about dietary breakfast habits in Spain.

The International Breakfast Research Initiative (IBRI) was established to provide evidence-based nutrient recommendations at breakfast, by assessing breakfast consumption in representative dietary surveys of the following six countries: Canada, Denmark, France, Spain, UK and the US [9]. Within this context and considering the recent ANIBES Study (Anthropometric data, macronutrients and micronutrients intake, practice of physical activity, socioeconomic data and lifestyles in Spain) [15] national dietary survey, the objectives of the current study in Spain are to present breakfast consumption patterns, nutrients and food group intakes at breakfast and their contribution to daily intakes in a representative sample of the Spanish population (9–75 years). The relationship between nutrient and food group intakes at breakfast and overall diet quality using the Nutrient Rich Food Index 9.3 scoring method, is also examined according to age [9].

## 2. Materials and Methods

### 2.1. Population

This study was based on data derived from the ANIBES Study, a nationally representative survey conducted in the Spanish population aged 9–75 years; the design and methodology of the ANIBES Study has been described in detail elsewhere [15]. The final protocol was approved by the Ethical Committee for Clinical Research of the Region of Madrid (Spain). Written informed consent was obtained from all subjects. All data were collected by trained interviewers. Briefly, the ANIBES study was performed to record food and beverage intake, the dietary habits and the anthropometric data, as well as the energy expenditure and physical activity patterns of the Spanish population. The target population consisted of all inhabitants living in Spain (excluding the autonomous cities of Melilla and Ceuta in the north of Africa), aged nine to 75 years and living in municipalities of at least 2000 inhabitants. Specifically, the starting sample contained 2009 people between nine and 75 years old (±2.23% error and 95.5% confidence interval). The distribution by sex (50.4% men and 49.6% women) reflected the distribution of men and women in the country. The sample size for younger Spaniards (9–12 and 13–17 years old) was increased to correctly represent these age groups (±6.9% error). The random sample plus additional participants included 2285 participants. The fieldwork for the ANIBES study was completed over the course of three months, between September and November 2013, after two pilot studies had been carried out (June–September 2013).

### 2.2. Intake of Energy, Nutrients and Foods

Dietary intake was assessed by a 3-day dietary record using a tablet device (Samsung Galaxy Tab 27.0, Korea) on two weekdays and one weekend day. The dietary recall consisted of describing every food item and beverage consumed during the day and taking a picture of the meal plate before and after consuming it, including the information on all foods and beverages that were consumed at home and away, as well as eating habits (e.g., recipes, brands, types of milk and fat spreads usually consumed, among other data). Participants who declared or demonstrated that they were unable to use the tablet device (21%) (mainly the elderly people, 65–75 years) were offered other options, such as using a digital camera and a paper record, and/or telephone interviews. A manual of procedures to facilitate food collection and additionally, a central server was arranged to facilitate the interviewers, encoders and nutritionist-dieticians. Because of this technology, it was possible in the ANIBES study to verify and codify the information compiled from each participant almost in real time.

Food, beverages and energy and nutrient intake were calculated using software (VD-FEN 2.1, Madrid, Spain) that was newly developed for the ANIBES study by the Spanish Nutrition Foundation and which is based mainly on expanded and updated Spanish food composition tables [16]. Finally, the data were grouped into 16 food groups, 29 subgroups and 761 ingredients for subsequent analysis [15].

### 2.3. Regularity of Breakfast Consumption

Breakfast has been considered as the first intake declared by the participant after overnight sleeping.

The regularity of breakfast was defined as follows:

- Breakfast on each of the three study days: Regular consumer
- Breakfast on two of the study days: Irregular consumer
- Breakfast on just one or none of the days of the study: Skippers & no breakfast

It was decided to combine the groups consuming breakfast on just one of the study days with those that never consumed a breakfast, given the very low numbers overall (1.44% of skippers and 3.44% take breakfast only one day) (see Table 1).

### 2.4. Calculation of Dietary Quality of the Diet by NRF 9.3

The previously published and validated Nutrient Rich Foods (NRF) index was the principal measure of nutrient density for the total diet [17,18]. The NRF 9.3d variant is an energy-adjusted diet quality score that is based on nine qualifying nutrients and three disqualifying nutrients. Therefore, the present applied version was based on the sum of the percentage of the daily values for nine nutrients that were encouraged in consumption (protein, dietary fibre, vitamin A, vitamin C, vitamin E, calcium, iron, magnesium and potassium), minus the sum of the percentage of maximum recommended values for three nutrients that were recommended for limited consumption (saturated fat, total or added sugar and sodium); all daily values were calculated per 2000 kcal and capped at 100%. As used in the IBRI studies, the NRF 9.3 score reflects daily nutrient intakes (normalized to an intake of 2000 kcal), expressed as the percentages of the national daily values for food labelling purposes. Vitamin E was replaced by vitamin D in the list of the nine nutrients to encourage in consumption.

The algorithm for the index subtracts the sum of the three nutrients to limit from the sum of the nine nutrients to encourage in consumption, expressed as a multiple of 100:

(∑sub-scores positive × 100) − (∑sub-scores negative × 100)

For each nutrient, the sub-score was calculated. For the nutrients to encourage, sub-scores above 100 were truncated to 100 for that nutrient. For the nutrients recommended to limit, if the sub-score was less than 100, then 0 was assigned to the sub-score. The maximum possible score was 900 points, reflecting a diet where intakes per 2000 kcal were ≥ the daily values for the nine nutrients to encourage and were ≤ daily values for the three nutrients to limit. Similar, 600 points reflected a diet where intakes per 2000 kcal were ≥ the daily values for the nine nutrients to encourage and were ≥ the daily values for the three nutrients recommended to limit.

The NRF 9.3 scores were calculated on the basis of the EU reference daily values and the maximum recommended values for nutrients, based on a 2000-kcal diet. Positive and negative nutrient sub-scores were calculated for both children and adults. For added sugar, an upper limit of 10% of energy intake was used as a nutrient reference value (NRV) according to the recommendation by the World Health Organisation (WHO) [19]. Daily sodium intakes were converted to salt by applying a conversion factor of 2.5 and the NRV was 6 g (In this survey, the salt intake only came from natural foods and some packaged foods; manually added salt was not collected). The NRV for dietary fibre was defined as 25 g, based on EFSA recommendations [20]. For the European countries participating in the IBRI studies (Denmark, France, United Kingdom and Spain), the methodology of NFR 9.3 used was the same.

### 2.5. Statistical Analysis

The results were presented according to the frequency of breakfast consumption, analysing only the days that the participants consumed breakfast (total sample, 1980 participants and total by age, 2255 participants) (Table 1). The random sample is used to show total sample data and to compare between sexes. To compare age groups and sex in age groups, a booster sample was included in order to expand those age groups that were less commonly represented in the random sample.

The comparison between groups (intakes, social or socioeconomic factors) was performed by a Student’s *t*-test for independent samples with a 95% confidence interval. In addition, the Kolmogorov–Smirnoff normality test was used to test the normality of the distribution: random sample (*n* = 2009) and random + booster sample (*n* = 2285). When considering breakfast consumption days only, extreme intakes of energy and each nutrient or those ± 3 SD (Standard Deviation) from the average, were eliminated. Variables were normalized applying scale transformations (square root and logarithmic). Statistical analysis of the nutrients by ANOVA (ANalysis Of VAriance) and ANCOVA (ANalysis of COVAriance), adjusted by the energy (kcal/day), was also performed. The averages shown were back-transformed to their original scales. Data analysis was performed with SPSS version 24.0 software package (IBM Corp., Armonk, NY, USA) and Microsoft Excel 2013.

## 3. Results

### 3.1. Regularity of Breakfast Consumption

**Figure 1:**
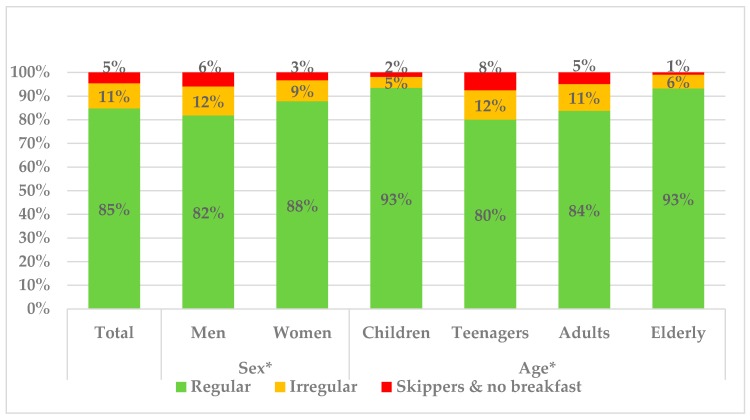
Regularity of breakfast consumption in the total ANIBES Spanish population, stratified by gender and age. * denotes statistical difference (*p* ≤ 0.05) by sex and age (ANOVA test).

Overall, the majority of the participants (84.8%) were regular breakfast consumers, while the remainder were either irregular (10.6%) or non-consumers (4.6%) (Figure 1). A higher proportion of women (87.9%) were regular breakfast consumers, compared with men (81.8%) (*p* = 0.05). After stratifying by age, the elderly (65–75 years) (93.2%) and children (9–12 years) (93.4%), were the groups who consumed breakfast more frequently, followed by adults (18–64 years) (83.8%) and teenagers (13–17 years) (80.1%). The highest proportion of irregular and non-breakfast consumers were clearly the adolescents (12.3%, 7.6%) respectively, representing one out of five consumers.

Even though no marked differences were seen in most age groups for breakfast habits when considering weekdays versus weekends, this was not the case for the teenagers group (13–17 years), for which a much higher regularity was observed during weekends (91% vs. 85%) but also a higher proportion of skippers, reaching a value of 10%. On the other hand, the regularity of breakfast consumption was not associated with the education level and income level of the main householder. Interestingly, the mean duration of breakfast consumption for the total population was 11.2 min and it varied according to regularity habits: regular breakfast consumers took on average 11.5 min, while irregular consumers and skippers took 9.13 and 9.11 min respectively (data not shown).

### 3.2. Contribution of Breakfast to Daily Energy and Nutrient Intakes

**Figure 2:**
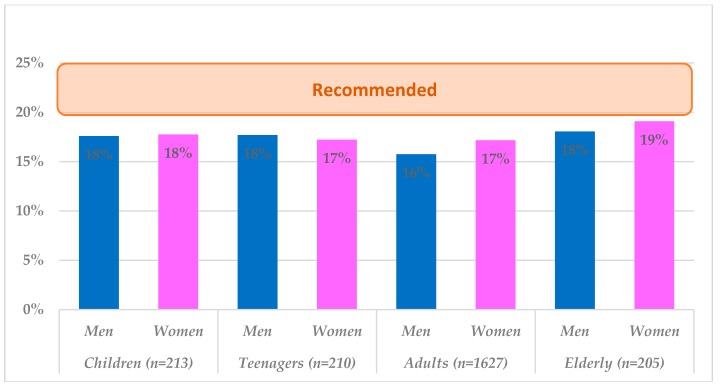
Contribution (%) of breakfast to the daily energy intake by age group and gender.

Breakfast contributed about 17% to the daily energy intakes in the total population and it varied by between 16–19% across gender and age. The data are summarized in Figure 2, according to dietary recommendations in Spain (20–25% of the total daily energy intake) [7,10].

The total carbohydrate contribution from breakfast for the national sample of the Spanish population was 53.6 ± 13.7%, higher than that which was obtained for the daily value (41.0 ± 7.1%), with no differences across the age groups. In general, sugar, whether added or total, contributed about two-fold more to breakfast energy intake that did all meals combined, for the total daily energy intake of such sugars. Considerable differences were seen for the contribution of added sugars at breakfast among children and teenagers (17.7 ± 8.0% and 17.2 ± 9.8% respectively), compared to the elderly. The opposite trend was shown for starch contribution, which was higher in the elderly.

**Figure 3:**
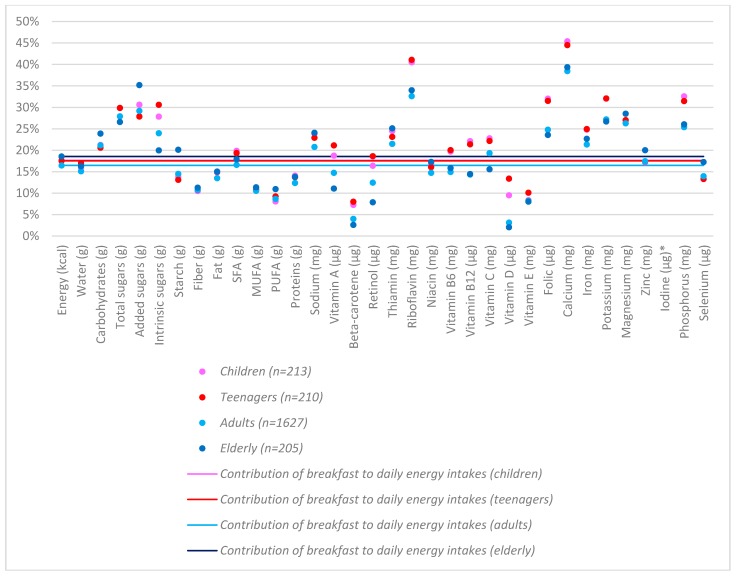
Contribution (%) of nutrient intakes at breakfast to daily nutrients intakes of Spanish breakfast consumers, by age group. The horizontal lines are the percentages of the daily energy intake consumed at breakfast, for the different age groups. * Iodine data was off the scale in the figure: 85% in children, 93% in adolescents, 84% in adults and 79% in the elderly.

As shown in Table 2 and Figure 3, relative to its daily energy contribution, breakfast contributed a higher proportion of daily carbohydrates, including total sugars, added sugars and intrinsic sugars but a lower proportion of daily starches, fibre and protein. Breakfast contributed a higher proportion of daily thiamin, riboflavin, folic acid, as well as iron, potassium, magnesium, phosphorus and calcium, across all of the age groups. On the other hand, the Spanish breakfast was low for vitamin D, vitamin E and beta-carotene. Breakfast contributed more to the intakes of calcium, riboflavin and phosphorus in children and teenagers, compared to adults. 

### 3.3. Contribution of Breakfast to Daily Food Groups Intakes in Spain

The percentage of consumers and the contribution of the food groups to the energy and nutrient intakes at breakfast for the total population is shown in Table 3. The food groups with the highest percentage of consumers were: milk and dairy products and grains and the most important were bread, non-alcoholic beverages and sugar and sweets. Other food groups were consumed by a remarkably lower percentage of individuals; for example, only 21% of the population were fruit consumers (higher for fresh fruit than fresh fruit juice) and only 12% consumed any kind of vegetables at breakfast.

The percentage of breakfast consumers for the major food groups, key subgroups and other food, as well as the contributions of breakfast to daily intakes by age group, are shown in Figures S1–S4. In the youngest age category (9–12 years), the highest frequency of consumption for the breakfast meal was chocolates (mainly as flavoured milk and powder), followed by bakery and pastry, whole milk and semi-skimmed milk. In contrast, the lowest percentage of consumers ate fresh fruit and cheeses, followed by skimmed milk and juices and nectars or olive oil. A very similar pattern was observed in the teenagers group (13–17 years). However, significant differences were observed with advancing age: in adults (18–64 years), the highest percentage of consumers drank coffee and infusions, followed by the consumption of sugar, bakery and pastry, semi-skimmed milk and bread. In this same age group, fruits, vegetables and olive oil were consumed by a remarkably higher percentage of consumers (13–25%) when compared to younger subjects. Finally, in the elderly (65–75 years), the highest consumption was reported for coffee and infusions, white bread, semi-skimmed milk and bakery and pastry; however, this age group also showed the highest consumption for fresh fruit and olive oil.

In terms of contribution from foods consumed at breakfast to energy and nutrient intake (Figures S1–S4), in children, energy was mainly provided by bakery and pastry, followed by whole milk and chocolates; in teens, the main food group contributing to consumption bakery and pastry and then whole milk and semi-skimmed milk. Somewhat different patterns were observed in the older groups, with a higher variety of foods providing consumed energy: in adults, bakery and pastry were the top foods consumed at breakfast but white bread, semi-skimmed milk, whole milk, butter/margarine, olive oil and chocolates (in descending order of importance) were also of importance; finally, in the elderly, even a heterogeneity was reported. Added sugars were mainly provided by chocolates (solids and liquids/powder), the bakery and pastry group and sugar, across all the age groups. As for calcium, milk in its different presentations was the main contributor (ranking 69–74% across the age groups). Of interest, breakfast cereals were an important source of vitamin D in the youngest groups.

### 3.4. Diet Quality (DQ): Nutrient and Food Group Intakes at Breakfast by Tertiles of Daily Dietary Quality

Breakfast consumers were divided into tertiles of daily dietary quality, based on the NRF 9.3 score. As expected, the mean daily NRF 9.3 score significantly increased from the lowest quality tertile (T1) to the highest (T3), in both children/teens and adults (*p* < 0.001) (Table 4). However, breakfast energy and total sugar intakes did not differ by the tertile of the DQ score in all age groups. In children, no differences according to DQ were observed for carbohydrates, monounsaturates and polyunsaturates. Intakes of added sugars, total fat and SFA, expressed as a percentage of breakfast energy intake, significantly decreased from the lowest to the highest DQ tertile (*p* < 0.001) in both children and adults. In contrast, protein, intrinsic sugars and fibre intake at breakfast showed the opposite trend in both age categories. In general, micronutrient intakes at breakfast also increased with an increasing DQ score in both children and adults (Table 5), particularly in the adults (with the exception of vitamin D), whereas no differences according to DQ were found for vitamins A and E, retinol and selenium in children. Sodium intake was unaffected by the diet quality score regardless of the age group.

The mean intakes of breakfast from food groups and subgroups and the proportions of children/teenagers and adults per tertile of foods consumed from each of the food groups and subgroups, are shown in Table S1. In children, milk and dairy product intake increased across the daily NRF 9.3 tertiles, as well as the tendency for a higher consumption of fruits in those classified in tertile 3 (reflecting the highest diet quality). Otherwise, few significant differences across the daily dietary quality tertiles that were observed for the other food groups were observed in this age group. Among adults, a higher number of foods impacted the quality of the diet: the intake of the milk and dairy products group (mainly cheeses, whole milk and yogurt/fermented milk), water, fruits and eggs increased across the tertiles of NRF 9.3 (the highest for those classified in tertile 3), while coffee/infusions, sugar and sweets (chocolates and sugar itself) decreased the tertiles (the lowest intake corresponded to the highest dietary quality tertile).

## 4. Discussion

Regular breakfast consumption is consistently associated with overall dietary quality and health outcomes, including lower body mass index (BMI) [21,22,23] and lower risk of cardiovascular disease [12,24,25]. However, there are currently evidence-based recommendations for optimal nutrient intakes at breakfast in Spain, although the dietary guidelines in our country have been recently updated including energy distribution throughout the day [11].

The present study has shown that the majority of the Spanish population are regular breakfast consumers, with a markedly higher frequency in women, which is also consistent with previous findings from the ANIBES Study in Spain reporting a better overall dietary pattern in women [1,26]. The prevalence of irregular breakfast consumption and breakfast skippers was found to be highest among teens (one in five). This is in accordance with previous studies conducted among the youngest in the Spanish population, where a significant decline in breakfast consumption has been observed during the last few years [27].

One of the main findings of the present study in Spain is that breakfast nutritional composition is nowadays unable to reach the recommended 20–25% of total daily energy intake across all age groups [11]. Other recent prospective studies in the Spanish population have reported similarly low energy intakes at breakfast [12,28]. It is imperative to understand that breakfast patterns in Spain are markedly different from other regions in the European Union [7,11,29]: people usually start with a light breakfast, typically with coffee, sweet rolls, toasts or biscuits, with no pancakes or similar foods, not too much meat, fried eggs, or even cereals for breakfast. In fact, data from the National Health Survey [30] reported that about 12% of the Spanish population just had a beverage (coffee, milk, tea, chocolate, etc.) for breakfast; about 50% of the population had usually a beverage combined with one solid food (bread, biscuits, pastries, or cereals), whereas only 10% of Spaniards consumed a more complete and adequate breakfast that included three or four food groups. The ALADINO Study, carried out with a national sample of Spanish boys and girls (6–9 years) showed that the most common breakfast consisted of milk or milk products (yogurt, smoothie, etc.) combined with a food from the cereals group (bread, toast, biscuits, or cereals) but only 2.8% had a quality breakfast comprising the three food groups (milk and/or derivative, cereals/derivatives and fruit) [31]; finally, in another representative study carried out in university students in Spain, it was shown that over 90% declared to eat or drink something for breakfast but only a 73% reported a breakfast that included at least 10% of the daily energy intake, whereas just a 36% had a breakfast with ≥ 20% of the total daily energy [32].

Overall, the Spanish breakfast can be considered to be a nutrient-dense meal that contributes considerably to the daily intake of micronutrients, highlighting the role of breakfast for calcium contribution, across all the age groups [33]. On the other hand, the Spanish breakfast shows a relatively poor nutrient density for vitamin D or beta-carotene. Recently, we reported that 76% and 79% of the population in Spain had intakes for calcium and magnesium below the 80% threshold of the national recommended daily intakes [34,35]; concerning the references at a Europe-wide level, these figures accounted for 66% and 72%, respectively [20]. As for vitamin D, 94% of the population reported intakes of below 80% of the daily intake recommendations at a national level, a figure that represents 93% when referring to the European reference values [20]. In another very recent study, a limited number of ANIBES participants had adequate folate intakes, whereas vitamin B12 intakes were covered for practically the entire Spanish population [36]. The relative high micronutrient intakes at breakfast observed in the current study are likely to be at least partially driven by current Spanish voluntary fortification practices (mainly in milk and milk products and cereals), although a tendency to decrease the presence of fortified foods (e.g., calcium and folic acid) in the Spanish market in the last years has been reported [37,38]. Notably, fortified breakfast cereal consumption has been associated with a higher dietary adequacy, specifically in relation to B vitamins, vitamin D and iron, without the risk of exceeding the Tolerable Upper Intake Level intake [39] and also towards a better nutrient-dense breakfast model in Spain [40]. The contribution of total fats and monounsaturated fatty acids from breakfast are also below the thresholds when compared to the daily energy, particularly for polyunsaturated fatty acids (PUFA). Finally, the macronutrient intake and distribution in the Spanish population is far from the population reference intakes and nutritional goals, especially for children and teenagers [10,11,41].

The analysis of foods that are contributing to the breakfast composition show strengths and weaknesses when compared to the dietary guidelines for the Spanish population: interestingly, milk and dairy products are first place in the rank of the percentage of consumers, with a significant contribution to calcium intake (ranking 69–74% across the all the age groups), followed by grains, with the most frequently consumed item being bread, although this is still clearly insufficient in terms of providing whole grains [10,41]. On the other hand, the low level of fruit and vegetable consumption at breakfast is of concern and it clearly represents an open window for urgent improvement. In fact, we have very recently shown that for the adult Spanish population, the servings of fruits and vegetables per day is just above two, versus the recommended five servings a day [42]. This situation is even worse in the youngest groups with barely one serving per day. It may be somewhat surprising that a low frequency of fruit and vegetable consumption has been reported in a Mediterranean country such as Spain. However, similar results have also been shown in other recent prospective studies [3,32,43].

Like all countries participating in the IBRI [9], we used the NRF 9.3 score to stratify breakfast by daily dietary quality and to evaluate the composition of breakfasts consumed according to the classification of daily diet quality: high, moderate, or low. It is important to consider that unlike other indices of diet quality, the NRF 9.3 is based on the intake of nutrients instead of food groups. The latter can be considered as more appropriate, as classifications of food groups and subgroups vary considerably across the IBRI-participating countries [9]. This study is the first to investigate the relationship between nutrient intakes at breakfast and the overall diet quality in Spain, by using this scoring method. As expected, the mean NRF 9.3 score significantly increased from the lowest quality tertile (T1) to the highest (T3) in both children and adults. However, breakfast energy, total sugars, or sodium intakes did not differ by the tertile of the diet quality score in all age groups. Intakes of added sugars, total fat and SFA expressed as percentages of breakfast energy intake, significantly decreased from the lowest to the highest dietary quality tertile, in both children and adults. In contrast, protein, intrinsic sugars and fibre intake at breakfast showed the opposite trend in both age categories. Globally, micronutrient intakes at breakfast also significantly increased with increasing scores, particularly in the adults (with the exception of vitamin D).

In children, the intake of milk and dairy products when considering the whole population group increased across the tertiles of NRF 9.3, as well as the tendency to also be higher for fruits. Otherwise, few significant differences across the tertiles were observed in this age group. Among adults, more marked differences on how the different foods and subgroups may impact the diet quality were observed; the intake of the milk and dairy products group (mainly cheeses, whole milk and yogurt/fermented milk), water, fruits and eggs increased across the tertiles of NRF 9.3, while coffee/infusions, sugar and sweets (chocolates and sugar itself) decreased.

Taken together, first, our data suggest that the consumption of breakfasts that include milk and milk products, as well as fruit, contribute to a higher quality of the daily diet; second, efforts are urgently needed in the youngest age groups to improve the breakfast variety and quality, since marked differences are reported when compared to the adult populations.

The strengths of the study include the large nationally representative sample of the ANIBES Spanish population and the assessment of nutrient and food group intakes at all meals based on three days of dietary recording by using new technologies (i.e., tablets). It is worth mentioning that the strengths of the ANIBES study are its careful design, the protocol and the methodology used. Furthermore, validated questionnaires were used to collect information on food records and they have shown good reliability and reproducibility. A limitation of this study is its cross-sectional design, which provides evidence for associations but not for causal relationships. The frequency of breakfast has been established based on a 3-day meal record and it is possible that some subjects have not been correctly identified as “breakfast skippers” or “always have breakfast.” Another limitation of our study is the possibility of dietary underreporting, which could affect the energy intakes and the range of foods reported at breakfast [44].

## 5. Conclusions

The present study shows that most of the Spanish population have breakfast regularly, although skipping reaches one out of five adolescents. Breakfast provides 16–19% of the daily intake of energy, which is below the dietary guidelines, with the elderly having the best patterns. This does in fact reflect the existing data that has been published for breakfast habits in Spain. The Spanish breakfast is high in total carbohydrates, added sugars, sodium, thiamin, riboflavin, folates, iron, potassium, magnesium, phosphorus and especially in calcium. In contrast, it is low in water intake, protein, dietary fibre, total fat, polyunsaturated/omega-3 fatty acids, beta-carotene and vitamins E and D. The dietary quality of breakfast consumers, assessed using the NRF9.3d score for diets, showed that higher NRF9.3d tertiles were associated with a greater degree of consumption of nutrients and food groups of interest, especially in the adults. These results reinforce the importance of continuous nutritional education in the Spanish population, to promote the importance of an adequate breakfast and that children and adolescents should be the main targeted age groups.

## Figures and Tables

**Table 1:** ANIBES (anthropometric data, macronutrients and micronutrients intake, practice of physical activity, socioeconomic data and lifestyles in Spain) sample according to the frequency of breakfast consumption.

	Total	Sex		Age			
		Men	Women	Children	Teenagers	Adults	Elderly
Regular	1704	829	875	199	169	1387	192
Irregular	212	124	88	10	26	186	12
Skippers	64	39	25	4	15	54	1
TOTAL	1980	992	988	213	210	1627	205

Total sample: 1980 participants. Total by age: 2255 participants.

**Table 2:** Energy and nutrient intakes at breakfast and daily contribution (%) among Spanish breakfast consumers.

	Total Population (*n* = 1980)			Children (*n* = 213)			Teenagers (*n* = 210)			Adults (*n* = 1627)			Elderly (*n* = 205)		
	Breakfast	Daily	% Daily Intakes	Breakfast	Daily	% Daily Intakes	Breakfast	Daily	% Daily Intakes	Breakfast	Daily	% Daily Intakes	Breakfast	Daily	% Daily Intakes
**Energy (kcal)**	296	1777	**17%**	342	1936	**18%**	349	1989	**18%**	293	1782	**16%**	295	1591	**19%**
**Water (g)**	240.1	1573.3	**15%**	215.3	1353.3	**16%**	221.3	1303.7	**17%**	243.2	1608.7	**15%**	251.9	1544.3	**16%**
**Carbohydrates (g)**	38.4	180.8	**21%**	44.7	210.5	**21%**	45.3	219.9	**21%**	37.9	179.4	**21%**	38.2	159.7	**24%**
Total sugars (g)	20.2	72.8	**28%**	26.5	88.5	**30%**	25.7	86.2	**30%**	19.9	71.4	**28%**	18.5	69.5	**27%**
Added sugars (g)	8.5	29.1	**29%**	14.0	45.8	**31%**	13.3	47.6	**28%**	8.3	28.6	**29%**	6.2	17.6	**35%**
Intrinsic sugars (g)	9.5	39.9	**24%**	11.4	40.8	**28%**	11.0	36.0	**31%**	9.4	39.2	**24%**	9.8	49.2	**20%**
Starch (g)	15.5	105.3	**15%**	16.6	119.8	**14%**	17.2	131.1	**13%**	15.3	105.3	**15%**	17.6	87.5	**20%**
**Fibre (g)**	1.3	12.1	**11%**	1.2	11.5	**11%**	1.2	11.3	**11%**	1.3	12.1	**11%**	1.6	14.0	**11%**
**Total Fat (g)**	10.4	76.0	**14%**	12.4	83.7	**15%**	12.6	84.1	**15%**	10.3	76.5	**13%**	9.9	65.8	**15%**
SFA (g)	3.9	23.1	**17%**	5.6	28.1	**20%**	5.3	27.5	**19%**	3.8	23.2	**17%**	3.3	18.6	**18%**
MUFA (g)	3.5	32.8	**11%**	3.8	34.2	**11%**	3.7	34.3	**11%**	3.5	33.0	**11%**	3.4	29.9	**11%**
PUFA (g)	1.1	12.8	**9%**	1.1	13.7	**8%**	1.3	14.1	**9%**	1.1	13.0	**9%**	1.2	10.7	**11%**
**Protein (g)**	9.2	73.0	**13%**	10.8	76.5	**14%**	10.8	78.6	**14%**	9.1	73.4	**12%**	9.2	66.3	**14%**
**Vitamin A (µg)**	34.3	236.3	**15%**	51.5	274.9	**19%**	50.6	239.5	**21%**	34.8	236.2	**15%**	24.6	222.0	**11%**
Beta-carotene (µg)	18.0	445.1	**4%**	26.2	360.4	**7%**	26.8	335.2	**8%**	18.1	450.8	**4%**	14.0	532.9	**3%**
Retinol (µg)	13.9	115.7	**12%**	27.1	165.4	**16%**	25.8	138.5	**19%**	14.2	114.2	**12%**	7.5	95.4	**8%**
**Thiamine (mg)**	0.17	0.75	**22%**	0.20	0.82	**25%**	0.20	0.85	**23%**	0.16	0.76	**22%**	0.17	0.67	**25%**
**Riboflavin (mg)**	0.33	0.98	**33%**	0.46	1.13	**41%**	0.43	1.06	**41%**	0.32	0.98	**33%**	0.32	0.94	**34%**
**Niacin (mg)**	3.1	20.7	**15%**	3.5	20.6	**17%**	3.5	21.9	**16%**	3.1	21.0	**15%**	3.2	18.2	**17%**
**Vitamin B_6_ (mg)**	0.14	0.94	**15%**	0.19	0.95	**20%**	0.20	0.98	**20%**	0.14	0.94	**15%**	0.14	0.90	**16%**
**Vitamin B_12_ (µg)**	0.45	3.01	**15%**	0.66	2.97	**22%**	0.59	2.75	**21%**	0.43	3.03	**14%**	0.43	2.96	**14%**
**Vitamin C (mg)**	5.2	28.1	**19%**	5.7	24.9	**23%**	4.9	22.0	**22%**	5.5	28.3	**19%**	5.4	34.5	**16%**
**Vitamin D (µg)**	0.0	1.4	**3%**	0.1	1.1	**10%**	0.1	1.1	**13%**	0.0	1.4	**3%**	0.0	1.4	**2%**
**Vitamin E (mg)**	0.3	3.7	**8%**	0.3	4.0	**8%**	0.4	4.1	**10%**	0.3	3.8	**8%**	0.2	3.1	**8%**
**Folates (µg)**	21.5	85.8	**25%**	29.4	91.7	**32%**	27.9	88.6	**32%**	21.3	85.8	**25%**	19.7	83.5	**24%**
**Calcium (mg)**	196.9	502.0	**39%**	285.0	627.6	**45%**	260.1	584.2	**45%**	189.6	493.0	**38%**	186.9	474.5	**39%**
**Iron (mg)**	1.5	6.7	**22%**	1.8	7.1	**25%**	1.8	7.2	**25%**	1.4	6.7	**21%**	1.4	6.3	**23%**
**Potassium (mg)**	410.4	1491.6	**28%**	503.5	1570.7	**32%**	482.7	1504.0	**32%**	404.3	1487.1	**27%**	405.5	1517.6	**27%**
**Magnesium (mg)**	37.8	142.8	**26%**	38.7	146.8	**26%**	38.8	143.4	**27%**	37.7	143.2	**26%**	40.1	140.4	**29%**
**Zinc (mg)**	1.0	5.9	**18%**	1.1	6.1	**17%**	1.1	6.3	**17%**	1.0	6.0	**17%**	1.1	5.4	**20%**
**Iodine (µg)**	115.6	135.9	**85%**	160.2	188.0	**85%**	148.2	158.7	**93%**	110.7	131.2	**84%**	114.2	144.0	**79%**
**Selenium (µg)**	7.9	55.6	**14%**	7.6	57.2	**13%**	7.9	59.4	**13%**	7.8	55.8	**14%**	8.8	50.9	**17%**
**Phosphorus (mg)**	217.7	835.5	**26%**	302.8	929.6	**33%**	282.9	898.9	**31%**	211.7	833.5	**25%**	203.0	779.6	**26%**
**Sodium (mg)**	295.2	1383.6	**21%**	369.7	1548.7	**24%**	363.2	1584.0	**23%**	288.0	1385.5	**21%**	284.9	1182.8	**24%**

Note: Only days where breakfast is consumed are considered. Extreme intakes, or those ± 3 SD from the average were eliminated. Variables were normalized, applying scale transformations (square root and logarithmic). The averages shown were back-transformed to their original scales. SFA: Saturated fatty acids; MUFA: monounsaturated fatty acids; PUFA: polyunsaturated fatty acids. Children: 9–12 years; Teenagers: 13–17 years; Adults: 18–64 years; Elderly: 65–74 years.

**Table 3:** Breakfast contribution of food groups and subgroups to energy and nutrient intakes in the total Spanish population (9–75 years).

	*n* = 1980	% of Consumers	Mean Intake (g)	Energy (kcal)	Total Sugars (g)	Added Sugars (g)	Fibre (g)	SFA (g)	Sodium (mg)	Vitamin A (µg)	Vitamin D (µg)	Folates (µg)	Calcium (mg)	Iron (mg)	Potassium (mg)	Phosphorus (mg)
**MILK AND DAIRY PRODUCTS**	1716	87%	155.4	84.5	8.1	0.7	0.0	1.9	98.2	27.3	0.0	8.1	208.0	0.2	237.7	158.2
Cheeses	201	10%	17.7	52.5	0.5	0.0	0.0	2.4	164.2	44.0	0.0	3.2	93.2	0.1	29.4	94.5
Milk	1599	81%	153.8	74.6	7.4	0.1	0.0	1.5	78.1	21.6	0.0	7.7	198.2	0.2	232.2	145.5
Semi-skimmed milk	854	43%	131.6	56.6	5.8	0.0	0.0	1.1	65.8	0.0	0.0	6.6	173.7	0.1	197.4	125.0
Skimmed milk	428	22%	139.0	48.7	7.0	0.0	0.0	0.1	72.3	0.0	0.0	7.0	180.7	0.1	208.5	132.1
Whole milk	585	30%	125.2	82.6	6.3	0.0	0.0	2.4	62.6	57.6	0.0	6.3	151.5	0.1	187.8	115.2
Other dairy products	61	3%	105.9	100.7	11.8	8.2	0.1	2.2	70.9	31.3	0.2	6.1	125.1	0.3	181.4	110.3
Yogurt and fermented milk	155	8%	69.9	58.0	7.9	3.6	0.0	1.1	40.7	10.5	0.0	3.9	88.3	0.1	127.0	84.5
**GRAINS**	1690	85%	44.7	159.3	4.3	3.7	1.6	1.4	184.7	9.5	0.2	14.7	29.0	1.1	57.4	56.7
Bakery and pastry	922	47%	34.5	151.3	5.6	5.3	1.2	2.4	112.2	16.9	0.1	6.6	27.2	0.6	44.2	48.8
Bread	1012	51%	33.1	92.5	0.7	0.2	1.1	0.1	175.8	0.0	0.0	4.7	14.6	0.6	41.6	35.1
White bread	859	43%	32.0	89.4	0.7	0.1	0.8	0.1	173.6	0.0	0.0	3.5	14.3	0.6	32.7	28.2
Whole bread	214	11%	24.1	62.1	0.5	0.2	1.8	0.1	119.2	0.1	0.0	7.3	9.8	0.6	58.7	47.2
Breakfast cereals and cereal bars	288	15%	22.5	85.8	4.8	4.2	0.9	0.2	103.7	0.0	1.0	43.4	27.4	1.9	9.9	22.6
Grains and flours	158	8%	18.5	66.5	0.1	0.0	1.2	0.1	5.9	0.1	0.0	9.1	7.6	0.7	46.6	53.1
**NON-ALCOHOLIC BEVERAGES**	1547	78%	139.2	15.7	3.0	0.7	0.0	0.0	5.4	2.6	0.0	3.6	6.1	0.2	85.6	10.6
Coffee and infusions	1228	62%	69.9	3.0	0.5	0.0	0.0	0.0	2.4	0.0	0.0	0.0	4.0	0.1	65.5	5.5
Juices and nectars	237	12%	112.8	52.4	12.5	0.7	0.1	0.0	5.7	16.9	0.0	14.0	11.7	0.4	160.8	15.2
Other drinks (non-alcohol)	124	6%	126.5	47.8	4.7	2.7	0.0	0.3	29.7	0.0	0.0	17.6	12.1	0.4	111.7	43.7
Water	489	25%	164.0	0.0	0.0	0.0	0.0	0.0	0.0	0.0	0.0	0.0	0.0	0.0	0.0	0.0
**SUGAR AND SWEETS**	1510	76%	11.6	42.1	8.7	8.4	0.0	0.3	39.1	0.5	0.0	1.6	3.2	0.2	65.4	31.8
Chocolates	611	31%	12.1	47.0	7.3	7.2	0.0	0.6	95.3	0.8	0.0	3.9	6.1	0.5	156.5	77.2
Jams and others	255	13%	13.6	36.5	9.1	7.5	0.1	0.0	2.7	1.0	0.0	0.0	3.0	0.0	8.8	2.5
Other sweets	257	13%	0.3	0.1	0.0	0.0	0.0	0.0	0.0	0.0	0.0	0.0	0.0	0.0	0.0	0.0
Sugar	863	44%	7.6	29.5	7.4	7.4	0.0	0.0	0.2	0.0	0.0	0.0	0.4	0.0	1.0	0.1
**OILS AND FATS**	841	42%	10.1	80.4	0.0	0.0	0.0	2.8	21.5	52.5	0.0	0.0	0.7	0.0	0.7	0.7
Butter, margarine and shortening	429	22%	12.2	89.4	0.0	0.0	0.0	4.2	42.1	102.2	0.1	0.0	1.5	0.0	1.5	1.3
Olive oil	475	24%	6.4	57.3	0.0	0.0	0.0	1.0	0.0	0.6	0.0	0.0	0.0	0.0	0.0	0.0
**FRUITS**	415	21%	123.9	64.5	11.3	0.0	1.7	0.2	3.0	20.8	0.0	22.3	19.8	0.4	225.5	27.7
Fresh fruit	289	15%	127.9	55.3	10.9	0.0	2.1	0.1	2.8	25.0	0.0	13.7	16.4	0.4	219.4	20.8
Fresh fruit juice	118	6%	109.1	46.9	10.9	0.0	0.1	0.0	1.1	10.9	0.0	40.4	16.9	0.2	181.1	20.7
**MEAT AND MEAT PRODUCTS**	386	19%	28.4	68.2	0.1	0.1	0.0	1.6	226.1	85.6	0.0	0.8	3.5	0.6	66.1	45.6
**VEGETABLES**	242	12%	19.0	4.2	0.6	0.0	0.3	0.0	3.2	25.1	0.0	4.7	2.9	0.1	50.1	5.2
**SAUCES AND CONDIMENTS**	215	11%	1.2	3.1	0.0	0.0	0.0	0.0	45.6	2.6	0.0	2.0	1.8	0.0	4.0	9.7
**EGGS**	136	7%	23.7	27.8	0.0	0.0	0.0	0.5	32.2	32.3	0.3	9.1	9.9	0.3	28.8	35.6

SFA: Saturated fatty acids.

**Table 4:** Breakfast energy and macronutrients intake of the Spanish population by tertile of the daily Nutrient-Rich Foods Index 9.3 (NRF 9.3) score, by age group.

	Children/Teenagers (9–17 Years)								Adults (18–75 Years)							
	T1 (Low)		T2 (Middle)		T3 (High)		*p*-Value	*p*-Value Adjusted for Daily Energy	T1 (Low)		T2 (Middle)		T3 (High)		*p*-Value	*p*-Value Adjusted for Daily Energy
	Mean	SD	Mean	SD	Mean	SD			Mean	SD	Mean	SD	Mean	SD		
**Daily NRF9.3 score**	**352.5**	**51.2**	**460.0**	**24.7**	**556.3**	**42.9**	**0.000**	**0.000**	**398.5**	**54.3**	**514.2**	**26.8**	**628.3**	**52.4**	**0.000**	**0.000**
Energy (kcal)	333	18	355	16	348	17	0.469	-	290	32	300	24	290	19	0.506	-
Water (g)	189	4	223	4	246	4	0.000	0.000	211	5	240	4	284	5	0.000	0.000
Carbohydrates (g)	42.6	2.2	45.7	2.0	46.9	2.4	0.177	0.029	36.3	3.6	38.3	3.1	39.2	2.7	0.059	0.000
Total sugars (g)	24.6	1.5	26.6	1.2	27.2	1.6	0.168	0.323	18.8	2.0	19.7	2.0	20.8	2.0	0.016	0.001
Added sugars (g)	14.5	1.6	14.1	1.4	12.4	1.6	0.137	0.008	9.9	2.3	8.4	2.3	6.2	2.0	0.000	0.000
Intrinsic sugars (g)	9.0	2.7	11.5	2.6	13.3	3.4	0.000	0.000	7.1	3.7	9.2	3.9	12.4	4.1	0.000	0.000
Starch (g)	15.4	3.4	17.3	2.5	18.0	2.5	0.251	0.168	14.7	4.5	15.8	3.8	16.1	3.0	0.229	0.008
Fibre (g)	0.95	0.34	1.37	0.33	1.34	0.31	0.007	0.007	1.01	0.48	1.27	0.49	1.76	0.57	0.000	0.000
Fat (g)	12.8	1.3	13.2	1.4	11.6	1.2	0.235	0.001	11.1	2.4	11.1	1.8	8.8	1.5	0.000	0.000
Saturates (g)	5.7	0.6	5.7	0.5	5.0	0.5	0.107	0.001	4.3	0.9	4.1	0.7	3.1	0.6	0.000	0.000
Monounsaturates (g)	3.8	0.5	3.9	0.5	3.6	0.5	0.640	0.091	3.7	0.9	3.8	0.7	3.0	0.7	0.000	0.000
Polyunsaturates (g)	1.25	3.20	1.31	3.48	1.04	3.01	0.307	0.128	1.18	4.15	1.28	3.71	0.95	3.47	0.003	0.000
Omega 3 fatty acids	0.08	2.01	0.09	2.30	0.07	2.21	0.556	0.470	0.07	2.68	0.07	2.54	0.05	2.70	0.008	0.002
Omega 6 fatty acids	1.06	3.18	1.10	3.51	0.83	3.00	0.185	0.055	0.92	4.28	0.91	3.88	0.63	3.45	0.000	0.000
Protein (g)	9.8	0.6	11.2	0.5	11.5	0.6	0.010	0.002	8.3	1.2	9.0	0.9	10.1	0.8	0.000	0.000
Carbohydrates (%)	52.2	9.5	52.6	9.9	54.7	10.3	0.081	0.058	53.0	15.3	52.6	13.4	55.3	13.1	0.001	0.004
Total sugars (%)	32.8	13.9	32.2	11.7	33.0	11.9	0.848	0.863	32.5	21.1	30.9	18.6	32.1	16.8	0.302	0.270
Added sugars (%)	19.7	10.5	17.3	8.0	15.3	7.6	0.000	0.000	18.5	17.2	14.6	12.9	11.5	10.9	0.000	0.000
Intrinsic sugars (%)	13.0	7.4	14.9	9.9	17.7	10.8	0.000	0.000	14.0	13.6	16.3	14.8	20.7	14.0	0.000	0.000
Starch (%)	19.4	10.8	20.4	9.8	21.6	10.1	0.175	0.208	20.5	12.4	21.7	11.6	23.2	12.1	0.000	0.000
Fat (%)	34.6	9.8	33.4	10.8	30.6	10.3	0.004	0.001	33.3	14.6	33.3	13.0	28.0	13.6	0.000	0.000
Saturates (%)	15.8	6.1	14.9	5.2	13.4	5.5	0.001	0.001	13.3	6.9	12.6	6.0	10.0	5.6	0.000	0.000
Monounsaturates (%)	10.3	4.1	10.0	3.9	9.5	4.2	0.276	0.131	11.1	6.1	11.6	6.5	10.2	6.7	0.001	0.002
Polyunsaturates (%)	4.8	5.3	4.7	5.3	3.9	4.0	0.204	0.155	5.5	6.5	5.7	6.1	4.6	5.4	0.005	0.015
Protein (%)	12.4	4.4	13.0	3.4	13.8	5.2	0.024	0.011	12.3	6.1	12.7	5.4	15.0	7.3	0.000	0.000

Note: only days of real consumption are considered. Extreme intakes or those ± 3 SD from the average, were eliminated. Variables were normalized applying scale transformations (square root and logarithmic). Statistical analysis of the nutrients for ANOVA and ANCOVA adjusted by the energy (kcal/day). The averages shown were back-transformed to their original scales.

**Table 5:** Breakfast micronutrient intake of the Spanish population by tertile of the daily Nutrient-Rich Foods Index 9.3 (NRF 9.3) NRF 9.3 score by age group.

	Children/Teenagers (9–17 years)								Adults (18–75 years)							
	T1 (Low)		T2 (Middle)		T3 (High)		*p*-Value	*p*-Value Adjusted for Daily Energy	T1 (Low)		T2 (Middle)		T3 (High)		*p*-Value	*p*-Value Adjusted for Daily Energy
	Mean	SD	Mean	SD	Mean	SD			Mean	SD	Mean	SD	Mean	SD		
**Daily NRF9.3 score**	**352.5**	**51.2**	**460.0**	**24.7**	**556.3**	**42.9**	**0.000**	**0.000**	**398.5**	**54.3**	**514.2**	**26.8**	**628.3**	**52.4**	**0.000**	**0.000**
Vitamin A (µg)	43.4	21.4	53.9	26.4	56.6	25.4	0.359	0.460	32.8	28.4	41.3	29.1	27.7	29.3	0.002	0.002
Beta-carotene (µg)	20.1	15.5	23.7	18.8	37.9	19.7	0.013	0.011	11.3	29.8	18.6	27.9	24.8	43.0	0.000	0.000
Retinol (µg)	23.9	29.4	29.8	34.1	26.0	36.4	0.740	0.946	17.2	33.1	19.4	36.8	6.5	33.6	0.000	0.000
Thiamine (mg)	0.15	1.39	0.20	1.40	0.25	1.49	0.000	0.000	0.13	1.54	0.16	1.49	0.21	1.58	0.000	0.000
Riboflavin (mg)	0.35	1.42	0.48	1.40	0.52	1.49	0.000	0.000	0.25	1.51	0.32	1.51	0.40	1.57	0.000	0.000
Niacin (mg)	2.9	2.0	3.6	2.0	4.1	2.2	0.000	0.000	2.6	2.4	3.0	2.3	3.7	2.5	0.000	0.000
Vitamin B6 (mg)	0.14	1.51	0.20	1.58	0.26	1.73	0.000	0.000	0.09	1.84	0.14	1.76	0.21	1.95	0.000	0.000
Vitamin B12 (µg)	0.47	0.09	0.67	0.08	0.74	0.08	0.000	0.000	0.35	0.10	0.45	0.12	0.51	0.10	0.000	0.000
Vitamin C (mg)	3.0	5.3	5.3	4.8	8.5	7.4	0.000	0.000	2.8	7.5	5.2	7.8	10.0	9.2	0.000	0.000
Vitamin D (µg)	0.07	3.17	0.10	3.54	0.24	3.42	0.000	0.000	0.03	3.33	0.05	3.78	0.05	4.36	0.164	0.190
Vitamin E (mg)	0.40	2.86	0.34	2.69	0.38	2.46	0.642	0.307	0.26	3.28	0.35	2.97	0.30	3.06	0.022	0.029
Folates (µg)	19.5	5.1	30.4	5.4	38.3	5.8	0.000	0.000	14.4	7.8	21.1	7.2	29.4	8.6	0.000	0.000
Calcium (mg)	224	20	294	15	304	19	0.000	0.000	161	25	187	23	221	19	0.000	0.000
Iron (mg)	1.50	1.82	1.85	1.90	2.01	2.00	0.000	0.000	1.22	2.15	1.42	2.12	1.66	2.20	0.000	0.000
Potassium (mg)	426	26	516	27	541	29	0.000	0.000	337	30	404	31	477	33	0.000	0.000
Magnesium (mg)	33.7	3.4	40.5	3.6	42.3	3.4	0.000	0.000	31.2	4.7	36.8	4.6	46.6	5.0	0.000	0.000
Zinc (mg)	0.97	0.10	1.10	0.08	1.15	0.10	0.046	0.035	0.93	0.19	1.06	0.15	1.17	0.14	0.000	0.000
Iodine (µg)	127	85	167	88	169	84	0.000	0.000	93	77	112	82	128	81	0.000	0.000
Phosphorus (mg)	261	16	314	18	305	17	0.003	0.003	185	23	212	21	236	18	0.000	0.000
Selenium (µg)	7.3	1.0	7.9	0.9	8.1	1.1	0.488	0.644	7.2	1.8	8.2	1.7	8.3	1.4	0.008	0.000
Sodium (mg)	343	34	372	27	385	32	0.227	0.254	277	62	293	49	293	36	0.414	0.139

Note: Only days of real consumption are considered. Extreme intakes or those ± 3 SD from the average, were eliminated. Variables were normalized by applying scale transformations (square root and logarithmic). Statistical analysis of the nutrients by ANOVA and ANCOVA were adjusted by energy (kcal/day). The averages shown were back-transformed to their original scales.

## Supplementary Materials

The following are available online at http://www.mdpi.com/2072-6643/10/9/1324/s1, Table S1: Mean food group intake at breakfast and proportions consuming food groups at breakfast by the daily Nutrient-Rich Foods Index 9.3 tertile among Spanish children/teenagers and adults. Figure S1: Breakfast Consumers (Children 9–12 years) for Food Groups and Subgroups (A) and Contribution of Breakfast to Daily Total Intake, Energy and Nutrients (B), Figure S2: Breakfast Consumers (Teenagers 13–17 years) for Food Groups and Subgroups (A) and Contribution of Breakfast to Daily Total Intake, Energy and Nutrients (B), Figure S3: Breakfast Consumers (Adults 18–64 years) for Food Groups and Subgroups (A) and Contribution of Breakfast to Daily Total Intake, Energy and Nutrients (B), Figure S4: Breakfast Consumers (Elderly 65–75 years) for Food Groups and Subgroups (A) and Contribution of Breakfast to Daily Total Intake, Energy and Nutrients (B).
