# Supplementary material for: Breakfast Consumption in Spain: Patterns, Nutrient Intake and Quality. Findings from the ANIBES Study, a Study from the International Breakfast Research Initiative
Source: Nutrients. 2018 Sep 18;10(9):1324. doi: 10.3390/nu10091324 (PMC6165504; doi:10.3390/nu10091324)
Supplement: Supplementary file 1 [file nutrients-10-01324-s001.zip › Figure S2 Ruiz E et al.docx]

**Figure S2.** Breakfast Consumers (Teenagers 13-17 years) for Food Groups and Subgroups (A), and Contribution of Breakfast to Daily Total Intake, Energy and Nutrients (B).
