# Supplementary material for: Breakfast Consumption in Spain: Patterns, Nutrient Intake and Quality. Findings from the ANIBES Study, a Study from the International Breakfast Research Initiative
Source: Nutrients. 2018 Sep 18;10(9):1324. doi: 10.3390/nu10091324 (PMC6165504; doi:10.3390/nu10091324)
Supplement: Supplementary file 1 [file nutrients-10-01324-s001.zip › Table S1 RuiZ E et al.docx]

**Table S1.** Mean food group intake at breakfast and proportions consuming food groups at breakfast by the daily Nutrient-Rich Foods Index 9.3 tertile among Spanish children/teenagers and adults.

|  | **Children (9-17)** | | | | | | | **Adults (18-75)** | | | | | | |
| --- | --- | --- | --- | --- | --- | --- | --- | --- | --- | --- | --- | --- | --- | --- |
|  | **T1 (n=141)** | | **T2 (n=140)** | | **T3 (n=142)** | |  | **T1 (n=141)** | | **T2 (n=140)** | | **T3 (n=142)** | |  |
|  | Mean (SD) | %consumers | Mean (SD) | %consumers | Mean (SD) | %consumers | **P-value** | Mean (SD) | %consumers | Mean (SD) | %consumers | Mean (SD) | %consumers | **P-value** |
| **MILK AND DAIRY PRODUCTS** | **145.6** | **89%** | **187.1** | **95%** | **186.8** | **95%** | **0.012** | **100.0** | **83%** | **124.2** | **85%** | **137.0** | **90%** | **0.000** |
|  | **(4.0)** |  | (**3.0)** |  | (**3.5)** |  |  | (**4.6**) |  | **(4.4**) |  | **(4.1**) |  |  |
| Cheeses | 14.1 | 6% | 15.7 | 9% | 10.4 | 13% | 0.009 | 11.5 | 10% | 13.5 | 9% | 14.4 | 12% | 0.009 |
|  | (2.5) |  | (3.3) |  | (2.0) |  |  | (2.9) |  | **(**2.5) |  | **(**2.8) |  |  |
| Milk | 148.9 | 79% | 174.9 | 90% | 179.3 | 92% | 0.112 | 104.7 | 75% | 123.9 | 80% | 137.3 | 85% | 0.000 |
|  | (3.5) |  | (3.4) |  | (3.5) |  |  | (4.1) |  | **(**4.1) |  | **(**3.8) |  |  |
| Semi-skimmed milk | 120.7 | 37% | 150.4 | 44% | 142.6 | 44% | 0.460 | 85.8 | 41% | 96.9 | 45% | 110.3 | 44% | 0.265 |
|  | (4.2) |  | (4.6) |  | (4.3) |  |  | (4.6) |  | **(**5.0) |  | (4.7) |  |  |
| Skimmed milk | 98.1 | 9% | 135.2 | 15% | 166.6 | 15% | 0.534 | 92.4 | 13% | 107.2 | 20% | 126.5 | 35% | 0.262 |
|  | (5.0) |  | (3.7) |  | (2.9) |  |  | (4.7) |  | **(**4.0) |  | (3.7) |  |  |
| Whole milk | 144.4 | 43% | 144.4 | 45% | 146.5 | 47% | 0.130 | 84.6 | 33% | 84.6 | 32% | 94.3 | 19% | 0.017 |
|  | (3.5) |  | (3.5) |  | (5.2) |  |  | (4.5) |  | **(**6.0) |  | (5.4) |  |  |
| Other dairy products | 77.8 | 11% | 111.6 | 7% | 80.5 | 6% | 0.597 | 62.0 | 5% | 68.2 | 3% | 130.7 | 0% | 0.231 |
|  | (2.7) |  | (3.2) |  | (6.3) |  |  | (3.3) |  | **(**6.2) |  | (3.9) |  |  |
| Yogurt and fermented milk | 58.9 | 7% | 57.1 | 11% | 61.4 | 8% | 0.945 | 39.5 | 6% | 48.8 | 8% | 57.4 | 10% | 0.007 |
|  | (4.0) |  | (3.8) |  | (3.7) |  |  | (5.8) |  | **(**4.6) |  | (3.7) |  |  |
| **GRAINS** | **36.7** | **82%** | **36.4** | **92%** | **35.7** | **93%** | **0.158** | **39.1** | **80%** | **38.0** | **86%** | **35.5** | **89%** | **0.002** |
|  | (**3.1**) |  | (**2.8**) |  | (**2.8**) |  |  | (**3.1**) |  | **(3.0**) |  | (**2.7**) |  |  |
| Bakery and pastry | 30.2 | 57% | 29.5 | 63% | 25.3 | 51% | 0.625 | 29.7 | 52% | 27.0 | 47% | 24.1 | 38% | 0.372 |
|  | (3.5) |  | (3.0) |  | (3.0) |  |  | (3.3) |  | **(**3.2) |  | (2.7) |  |  |
| Bread | 22.2 | 38% | 19.7 | 40% | 22.5 | 44% | 0.663 | 27.7 | 46% | 27.5 | 54% | 25.8 | 58% | 0.216 |
|  | (3.0) |  | (2.7) |  | (3.3) |  |  | (3.0) |  | **(**3.2) |  | (3.0) |  |  |
| White bread | 21.0 | 38% | 19.1 | 35% | 22.8 | 42% | 0.454 | 26.7 | 42% | 27.2 | 46% | 23.5 | 44% | 0.480 |
|  | (3.0) |  | (2.3) |  | (3.2) |  |  | (3.1) |  | **(**3.2) |  | (3.1) |  |  |
| Whole bread | 12.3 | 2% | 29.3 | 4% | 14.0 | 1% | 0.820 | 16.4 | 7% | 18.4 | 10% | 21.1 | 18% | 0.078 |
|  | (2.3) |  | (5.1) |  | (4.1) |  |  | (3.0) |  | **(**3.2) |  | (2.7) |  |  |
| Breakfast cereals and cereal bars | 14.4 | 17% | 16.1 | 29% | 18.4 | 42% | 0.256 | 14.1 | 6% | 16.4 | 11% | 19.4 | 21% | 0.632 |
|  |  |  |  |  |  |  |  |  |  |  |  | (3.1 |  |  |
|  | (2.2) |  | (2.9) |  | (2.8) |  |  | (2.3) |  | **(**3.0) |  |  |  |  |
| Grains and flours | 7.5 | 5% | 15.6 | 8% | 12.2 | 8% | 0.250 | 12.8 | 7% | 12.7 | 8% | 13.9 | 9% | 0.928 |
|  | (1.9) |  | (2.6) |  | (3.6) |  |  | (4.0) |  | **(**3.5) |  | (4.3)) |  |  |
| **NON ALCOHOLIC BEVERAGES** | **89.1** | **40%** | **87.1** | **28%** | **95.8** | **35%** | **0.878** | **87.4** | **82%** | **83.4** | **83%** | **86.8** | **85%** | **0.721** |
|  | (**5.5**) |  | (**6.9** |  | (**6.6**) |  |  | (**7.7)** |  | **(9.5**) |  | (**11.7**) |  |  |
| Coffee and infusions | 27.0 | 8% | 16.8 | 6% | 33.8 | 9% | 0.667 | 48.7 | 65% | 37.9 | 69% | 33.4 | 71% | 0.000 |
|  | (7.4) |  | (13.0) |  | (15.6) |  |  | (7.7) |  | **(**9.4) |  | (10.9) |  |  |
| Juices and nectars | 74.8 | 10% | 81.6 | 9% | 87.4 | 16% | 0.509 | 85.0 | 12% | 92.7 | 11% | 101.8 | 13% | 0.453 |
|  | (2.4) |  | (3.8) |  | (3.1) |  |  | (3.0) |  | **(**3.1) |  | (3.3) |  |  |
| Other drinks (non-alcohol) | 89.5 | 9% | 103.9 | 3% | 99.8 | 4% | 0.785 | 87.0 | 5% | 104.5 | 8% | 106.0 | 7% | 0.005 |
|  | (4.2) |  | (9.5) |  | (3.8) |  |  | (2.4) |  | **(**5.1) |  | (4.4) |  |  |
| Water | 113.5 | 13% | 107.8 | 14% | 94.2 | 14% | 0.686 | 112.2 | 24% | 120.1 | 25% | 140.2 | 30% | 0.019 |
|  | (3.7) |  | (3.8) |  | (5.2) |  |  | (5.0) |  | **(**4.5) |  | (5.)0 |  |  |
| **SUGAR AND SWEETS** | **12.9** | **78%** | **12.4** | **81%** | **11.7** | **79%** | **0.119** | **8.0** | **78%** | **7.5** | **79%** | **5.5** | **72%** | **0.052** |
|  | (**2.8**) |  | (**3.3**) |  | (**2.7**) |  |  | (**3.9**) |  | **(4.5**) |  | (**5.6**) |  |  |
| Chocolates | 11.5 | 70% | 12.5 | 69% | 10.9 | 70% | 0.081 | 9.1 | 29% | 9.6 | 29% | 8.7 | 21% | 0.003 |
|  | (2.5) |  | (2.4) |  | (2.3) |  |  | (2.6) |  | **(**2.5) |  | (2.5) |  |  |
| Jams and others | 12.2 | 6% | 10.8 | 9% | 10.5 | 7% | 0.783 | 11.0 | 10% | 10.8 | 15% | 11.9 | 17% | 0.349 |
|  | (1.7) |  | (2.1) |  | (1.6) |  |  | (2.7) |  | **(**2.3) |  | (x) |  |  |
| Other sweets | 10.0 | 1% | 0.2 | 2% | 0.0 | 1% | 0.604 | 0.1 | 9% | 0.2 | 14% | 0.2 | 20% | 0.317 |
|  | (1.2) |  | (3.3) |  | (0.0) |  |  | (1.9) |  | **(**2.4) |  | (2.2) |  |  |
| Sugar | 6.2 | 26% | 5.3 | 24% | 4.2 | 27% | 0.485 | 6.5 | 54% | 6.3 | 47% | 5.6 | 37% | 0.008 |
|  | (2.3) |  | (2.8) |  | (3.0) |  |  | (2.4) |  | **(**2.5) |  | (2.4) |  |  |
| **OILS AND FATS** | **8.6** | **26%** | **8.0** | **31%** | **6.5** | **32%** | **0.830** | **8.5** | **40%** | **8.3** | **48%** | **7.0** | **44%** | **0.630** |
|  | (**2.8**) |  | **(2.9**) |  | (**2.6**) |  |  | (**2.9**) |  | **(2.8**) |  | (**2.7**) |  |  |
| Butter, margarine and shortening | 12.3 | 16% | 10.4 | 21% | 8.6 | 15% | 0.510 | 10.8 | 23% | 9.6 | 26% | 9.0 | 18% | 0.368 |
|  | (2.0) |  | (2.5) |  | (2.1) |  |  | (2.6) |  | **(**2.7) |  | (2.6) |  |  |
| Olive oil | 4.6 | 8% | 4.1 | 9% | 4.7 | 17% | 0.875 | 4.8 | 21% | 5.5 | 27% | 5.1 | 29% | 0.985 |
|  | (2.8) |  | (2.3) |  | (2.6) |  |  | (2.3) |  | **(**2.3) |  | (2.3) |  |  |
| **FRUITS** | **55.0** | **7%** | **56.9** | **16%** | **88.2** | **23%** | **0.213** | **43.7** | **11%** | **70.2** | **21%** | **89.3** | **34%** | **0.040** |
|  | (**4.8**) |  | (**8.1**) |  | (**9.9**) |  |  | (**9.1)** |  | **(9.1**) |  | **10.0**) |  |  |
| Fresh fruit | 50.3 | 4% | 67.4 | 11% | 66.5 | 15% | 0.274 | 54.7 | 6% | 63.1 | 15% | 105.5 | 25% | 0.922 |
|  | (2.9) |  | (3.6) |  | (12.6) |  |  | (7.0) |  | **(**8.1) |  | (7.5) |  |  |
| Natural fruit juice | 109.9 | 2% | 98.3 | 4% | 108.6 | 11% | 0.715 | 76.1 | 4% | 86.0 | 7% | 100.5 | 8% | 0.855 |
|  | (2.5) |  | (3.3) |  | (2.9) |  |  | (2.6) |  | **(**3.4) |  | (3.8) |  |  |
| **MEAT AND MEAT PRODUCTS** | **23.1** | **16%** | **17.2** | **15%** | **18.6** | **16%** | **0.749** | **24.1** | **21%** | **20.3** | **20%** | **19.3** | **19%** | **0.311** |
|  | (**3.6**) |  | (**2.6**) |  | (**2.6**) |  |  | **(3.3)** |  | **(2.8**) |  | (**2.7**) |  |  |
| **VEGETABLES** | **11.9** | **5%** | **10.7** | **4%** | **23.7** | **6%** | **0.902** | **13.6** | **12%** | **10.9** | **13%** | **14.2** | **14%** | **0.029** |
|  | (**2.1**) |  | (**1.8**) |  | (**6.4**) |  |  | (**3.1**) |  | **(4.2**) |  | (**4.4**) |  |  |
| **SAUCES AND CONDIMENTS** | **0.3** | **4%** | **0.4** | **5%** | **0.4** | **8%** | **0.328** | **0.3** | **12%** | **0.3** | **12%** | **0.3** | **11%** | **0.532** |
|  | (**4.6**) |  | (**2.3**) |  | (**4.0**) |  |  | (**3.7**) |  | **(3.1**) |  | (**4.1**) |  |  |
| **EGGS** | **9.9** | **6%** | **20.6** | **8%** | **19.9** | **7%** | **0.764** | **12.7** | **8%** | **11.3** | **8%** | **18.3** | **6%** | **0.008** |
|  | (**4.3**) |  | (**3.6**) |  | (**7.0**) |  |  | (**4.2**) |  | **(4.9**) |  | (**5.8**) |  |  |

*Note: Only days of real consumption are considered. Extreme intakes or those +/-3 SD from the average, were eliminated. Variables were normalized applying scale transformations (square root and logarithmic). Statistical analysis of the nutrients for ANOVA and ANCOVA adjusted by the energy (kcal/day). The averages shown were back-transforming to their original scale*
